# Comprehensive Assessment of Potential Multiple Myeloma Immunoglobulin Heavy Chain V-D-J Intraclonal Variation Using Massively Parallel Pyrosequencing

**DOI:** 10.18632/oncotarget.469

**Published:** 2012-04-20

**Authors:** Renee C. Tschumper, Yan W. Asmann, Asif Hossain, Paul M. Huddleston, Xiaosheng Wu, Angela Dispenzieri, Bruce W. Eckloff, Diane F. Jelinek

**Affiliations:** ^1^ Department of Immunology, Mayo Clinic, Rochester, MN, USA; ^2^ Department of Health Sciences Research, Mayo Clinic, Rochester, MN, USA; ^3^ Department of Orthopedic Surgery, Mayo Clinic, Rochester, MN, USA; ^4^ Department of Internal Medicine, Mayo Clinic, Rochester, MN, USA; ^5^ Department of Biochemistry and Molecular Biology, Mayo Clinic, Rochester, MN, USA

**Keywords:** IGHV, multiple myeloma, heterogeneity, massively parallel sequencing

## Abstract

Multiple myeloma (MM) is characterized by the accumulation of malignant plasma cells (PCs) in the bone marrow (BM). MM is viewed as a clonal disorder due to lack of verified intraclonal sequence diversity in the immunoglobulin heavy chain variable region gene (IGHV). However, this conclusion is based on analysis of a very limited number of IGHV subclones and the methodology employed did not permit simultaneous analysis of the IGHV repertoire of non-malignant PCs in the same samples. Here we generated genomic DNA and cDNA libraries from purified MM BMPCs and performed massively parallel pyrosequencing to determine the frequency of cells expressing identical IGHV sequences. This method provided an unprecedented opportunity to interrogate the presence of clonally related MM cells and evaluate the IGHV repertoire of non-MM PCs. Within the MM sample, 37 IGHV genes were expressed, with 98.9% of all immunoglobulin sequences using the same IGHV gene as the MM clone and 83.0% exhibiting exact nucleotide sequence identity in the IGHV and heavy chain complementarity determining region 3 (HCDR3). Of interest, we observed in both genomic DNA and cDNA libraries 48 sets of identical sequences with single point mutations in the MM clonal IGHV or HCDR3 regions. These nucleotide changes were suggestive of putative subclones and therefore were subjected to detailed analysis to interpret: 1) their legitimacy as true subclones; and 2) their significance in the context of MM. Finally, we report for the first time the IGHV repertoire of normal human BMPCs and our data demonstrate the extent of IGHV repertoire diversity as well as the frequency of clonally-related normal BMPCs. This study demonstrates the power and potential weaknesses of in-depth sequencing as a tool to thoroughly investigate the phylogeny of malignant PCs in MM and the IGHV repertoire of normal BMPCs.

## INTRODUCTION

Multiple myeloma (MM) is a devastating and incurable plasma cell (PC) malignancy that has a median survival of 3-4 years following diagnosis and is the second most common hematologic malignancy in the United States [1, 2]. To improve overall patient outcomes, greater insight into disease etiology and progression is needed. Like normal B cells and PCs, the most discriminating molecular feature of MM cells is the immunoglobulin (Ig) molecule. The unique Ig molecular fingerprint is determined initially during B cell development when the Ig heavy chain variable region gene (IGHV) and the Ig light chain variable region gene (IGLV) undergo precise rearrangements. The diversity of the Ig molecular fingerprint can be further increased in B lineage cells that acquire Ig somatic hypermutations (SHM) during germinal center (GC) reactions. Memory B cells, which may undergo multiple GC expansions, often display particularly unique IGHV and IGLV sequences. The IGHV/IGLV sequences, therefore, serve as unparalleled identifiers of a given B cell or MM cell clone.

Within Ig molecules, the complementarity determining regions (CDRs) acquire the most SHM and thus diversity. The heavy chain CDR3 (HCDR3) serves as a unique clonal B cell marker because of a number of distinguishing features. Thus, the nucleotide and amino acid sequence of the HCDR3 are influenced by: 1) specific Ig heavy chain D (IGHD) and J region gene (IGHJ) usage; 2) junctional diversity introduced by IGHV-D-J rearrangement; 3) the precise IGHD gene reading frame; 4) addition of non-templated (N) and palindromic (P) nucleotides during B cell development; and 5) possible SHMs introduced following antigenic stimulation [3-9].

B cell malignancies exist at all stages of B cell development and differentiation. Elucidation of the molecular characteristics of rearranged IGHV genes and the HCDR3 region in particular may offer insight into the etiology of B cell malignancies as well as the history of malignant B lineage cells, including ongoing genetic evolution. For example, B cell malignancies exhibiting extensive IGHV intraclonal heterogeneity likely reflect transformation of B cells at the GC stage of differentiation that retain the ability to undergo SHM whereas those with homogeneous IGHV sequences may indicate post-GC malignant cells that can no longer activate the SHM process. Similarly, malignancies with limited intratumor IGHV heterogeneity may suggest ongoing genetic evolution resulting from multiple transits through GCs or non-conventional SHM occurring outside of GCs [10-12].

Thus, molecular IGHV analysis has been an area of intense study in B cell malignancies, most notably in chronic lymphocytic leukemia where there is a distinct bias of IGHV gene usage and the degree of SHM correlates with disease prognosis [13-15]. In contrast, less effort has been focused on analysis of MM Ig sequences [16-22]. Of relevance to this study, others have used IGHV sequence analysis to better understand the relationship between MM and its known premalignant condition monoclonal gammopathy of undetermined significance (MGUS) [23, 24] and have shown that while there is evidence for intraclonal heterogeneity in MGUS, MM PCs are homogeneous [17, 25-27]. This observation is compatible with the notion that disease progression to MM requires additional genetic changes that may only be gained by one of the “sister” clones initially generated during the GC response that gave rise to the abnormally sized PC expansion underlying the development of MGUS. However, these studies were carried out on a limited number of subclones for each patient leaving open the possibility that MM patients may harbor rare, but clonally related PCs potentially resulting from ongoing low level clonal evolution. Indeed, tumor heterogeneity is a common characteristic of cancer [28] and may reflect external influences or disordered regulatory mechanisms within the neoplastic cells. Furthermore, subtle genetic diversity within a clone may suggest a “branching” ancestral relationship [29] rather than the essentially linear concept of clonal evolution of tumor cells [30].

The advent of massively parallel sequencing has permitted extensive analysis of intraclonal heterogeneity especially as it relates to IGHV and T cell receptor sequences in both normal and malignant B and T cells [31-41]. To our knowledge, comprehensive sequencing methodologies have not yet been used to study MM. In this study, we exploited the power of massively parallel sequencing to better understand the phylogeny and potential for intraclonal heterogeneity of malignant PCs in MM as well as the effects of clonal expansion on the remaining non-malignant PC IGHV repertoire. For comparison purposes, we also analyzed normal bone marrow resident PCs (BMPCs). Lastly, we studied both genomic DNA and cDNA to determine which starting template better estimates the overall IGHV repertoire using deep sequencing methodologies.

## RESULTS

### Evaluation of clonal MM cells by conventional fluorescent sequencing

By direct sequencing and manual subcloning prior to 454 GS-FLX sequencing, the MM patient sample studied in this report was determined by the ImMunoGeneTics Information System (IMGT/V-Quest) software to express the IGHV3-74 gene with 18 somatic mutations (7.5%) as compared to the germline (GL) sequence. Furthermore, the IGHD2-2*01 (reading frame 2) and IGHJ4*02 genes were used with 11 N nucleotides but no P nucleotides in the HCDR3.

### Pyrosequencing using cDNA versus genomic DNA

**Figure 1:**
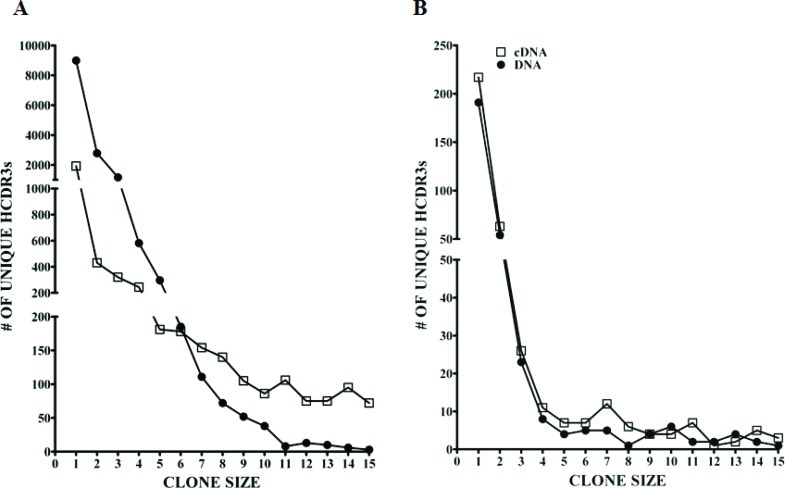
Ig clone size comparison of DNA versus cDNA as defined by unique HCDR3s The clone sizes based on unique HCDR3 are represented for (A) normal BMPCs and (B) normal residual non-tumor MM PCs after 454 GS-FLX sequencing. Open squares (□) represent cDNA and closed circles (•) represent DNA as the starting template. Only clones of size 1-15 sequences are shown.

The IGHV repertoire of the MM patient PCs and control BMPCs was analyzed in detail using massively parallel sequencing. It is known that PCs express high levels of Ig mRNA transcripts and the expression levels of Ig mRNA between individual PCs may vary significantly. Because this variance could lead to potential artifactual skewing of the IGHV repertoire, we compared the repertoire of IGHV sequences amplified from cDNA versus genomic DNA. Although the analysis of genomic DNA would overcome the expression bias of Ig genes in cDNA samples, it has the limitation of also capturing the non-productively rearranged IGHV sequences. In the context of MM, we found that the non-productively rearranged IGHV sequences were highly homologous to the dominant MM clone but suffered from insertions of nucleotides in homopolymer regions or had incomplete HCDR3s. After following the methodology which eliminated these sequences and others from further analysis, we obtained 30,426 and 24,717 IGHV sequences in the MM DNA and cDNA cohorts, respectively; whereas in the BMPC sample there were 26,559 DNA and 36,150 cDNA sequences (Table S1). IGHV sequences were then grouped into clones as defined by identical HCDR3 amino acid sequences and sorted on the basis of clone size. Figure 1 compares the number of unique HCDR3 sequences observed in normal BMPCs (Panel A) and normal residual non-tumor PCs in the MM patient (Panel B) when the starting template was cDNA or DNA. Only those HCDR3s with clone sizes ranging from 1-15 are shown. Figure 1A and Table S2 clearly demonstrate that there was a 4.7 fold increase in the number of HCDR3s represented by a single sequence in the DNA as compared to the cDNA using the BMPC samples. These data validate our hypothesis that a better estimate of Ig repertoire is achieved using genomic DNA in this type of analysis. Of note, the differences between cDNA and DNA were not as clearly defined in the MM PC sample most likely due to the extreme expansion of the malignant clone, which significantly decreased the overall number of IGHV sequence reads from non-tumor PCs.

### In-depth IGHV analysis of PCs isolated from a MM patient

**Figure 2:**
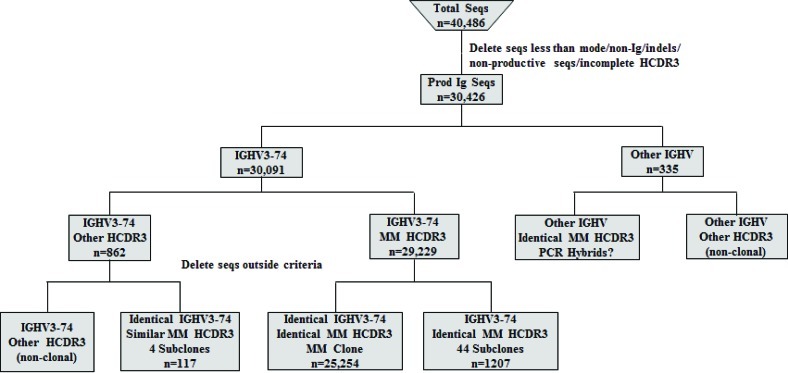
Algorithm for analyzing IGHV sequences (seqs) obtained from MM DNA sample After filtering the data, the remaining productive sequences were categorized by IGHV gene usage and those with the MM clone IGHV3-74 gene were studied in detail looking for putative subclones.

**Figure 3:**
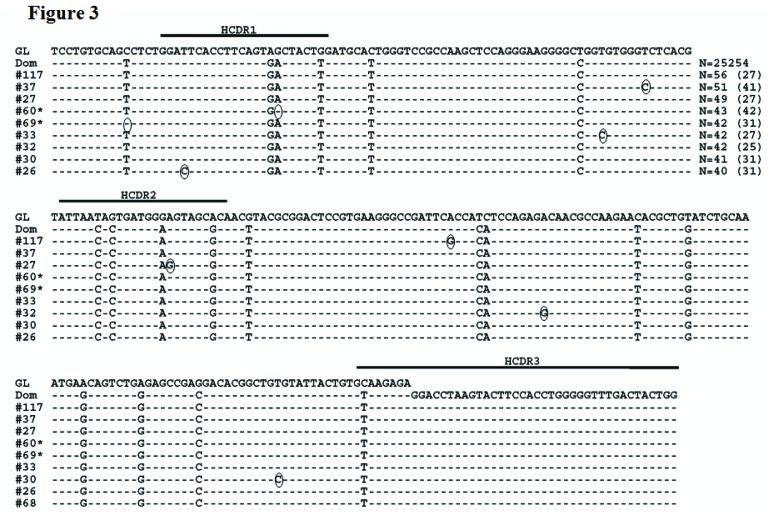
Sequence alignment of MM subclones using IGHV3-74 and the dominant clone HCDR3 The dominant MM clone (Dom) and the 9 largest representative subclone sequences with the dominant HCDR3 using the IGHV3-74 gene when DNA is the starting template are shown. Dashes indicate sequence homology with the IGHV3-74 GL sequence and nucleotide changes from the dominant clone are circled. Sequence numbers with an * indicate that the nucleotide deviations from the dominant clone reflect reversions back to GL. Values in parentheses represent the number of identical sequences found in cDNA.

**Figure 4:**
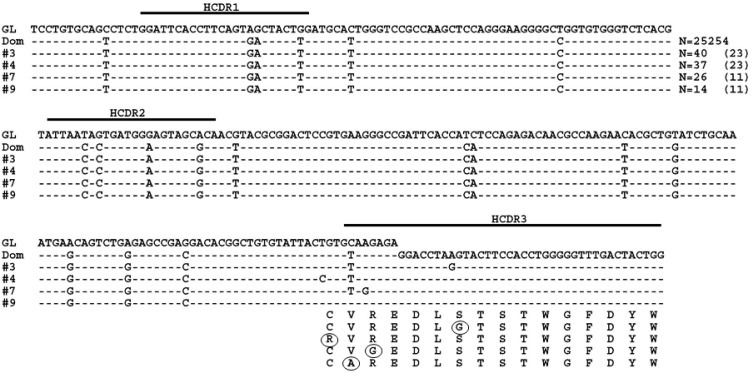
MM subclones using IGHV3-74 with non-synonymous HCDR3s Representative subclones with the identical IGHV gene as the dominant clone (Dom) but with HCDR3s that varied by 1 amino acid from the dominant sequence when DNA is the starting template are shown. Dashed lines indicate sequence homology with the IGHV3-74 GL sequence. Nucleotide deviations from the dominant clone are shown and amino acid changes are circled. Values in parentheses represent the number of identical sequences found in cDNA.

Detailed analysis was then done on the sequences generated from the MM patient. Both the DNA and cDNA cohorts were analyzed in a parallel manner with Figure 2 detailing our data filtering process when DNA was the starting template. As expected, the majority of productively rearranged sequences in both the DNA and cDNA libraries used the IGHV3-74 gene (30,091/30,426 productive Ig sequences or 98.9% in DNA; 23,790/24,717 productive Ig sequences or 96.2% in cDNA). The IGHV3-74 sequences were then grouped on the basis of HCDR3 amino acid sequence. The largest clonal amino acid HCDR3 sequence was VREDLSTSTWGFDY and 29,229 sequences were found using IGHV3-74 with this precise HCDR3 in the DNA cohort and 23,125 in the cDNA cohort. Within the DNA group, 25,254 sequences were identical in nucleotide sequences of the HCDR3 and IGHV regions (83% of the productive Ig sequences) and 18,889 were identical in the cDNA group (81.6% of the productive Ig sequences). Based on our findings that a more extensive IGHV repertoire emerged when the starting material was DNA, further analysis was focused on the remaining 3,975 out of 29,229 sequences in the DNA cohort that were highly likely to be clonally related to the dominant clone. To assess whether these sequences may represent MM subclones, we followed our algorithm to eliminate sequences with pyrosequencing errors whereby sequences with any differences from the dominant IGHV3-74 clone in or near homopolymers were excluded. Sets of identical sequences with sizes less than 10 (in both the DNA and cDNA samples) or consisting of sequences in only one orientation were also eliminated. All remaining sets of sequences had a verified Phred quality score greater than 35 including any deviations from the dominant clone, thereby significantly exceeding our threshold of 20. After applying this algorithm, 44 putative subclones (1207 sequences) were identified that displayed the dominant HCDR3 but with single nucleotide deviations in the IGHV region distinguishing them from the dominant clone (Figure 2). The number of sequences in each subclone varied from 13-56 in DNA and the 9 largest of these subclones are shown in Figure 3. As required by our algorithm, thorough analysis of the cDNA IGHV sequence cohort confirmed the presence of each of these subclones with at least 10 sequences. Of note, there were 2 subclones (#60 and #69) where the single deviation was not a result of an additional mutation but reflected the original GL sequence of IGHV3-74 at that particular nucleotide (Figure 3). Finally, there were 862 IGHV3-74 sequences that did not express an HCDR3 identical to the dominant MM clone. Using the same algorithm to identify subclones within these sequences, we discovered 4 additional MM-related subclones. These subclones (117 sequences) displayed IGHV regions identical to the dominant clone but HCDR3 regions that differed by a single nucleotide change resulting in an amino acid change in the HCDR3 (Figure 4).

We next focused on the small subset of sequences with IGHV designations other than IGHV3-74 (n=335; Figure 2). This group consisted of 36 other IGHV genes with a range of 1-20 unique HCDR3s in each IGHV gene (187 unique HCDR3s total; Figure S1; Table S3). Detailed analysis did not uncover any evidence of subclones within this group with the majority of the distinctive HCDR3s being represented by a single sequence. Of interest, we observed sequences that unexpectedly displayed an HCDR3 nucleotide and amino acid sequence that was identical to the dominant clone. These clones instead differed sufficiently within the IGHV region to be classified by IMGT/V-Quest as a different IGHV gene (IGHV3-23, 3-48, 3-30, 3-33 and 3-7). Despite being found in the cDNA as well, these sequences did not meet our criteria for subclone designation and most likely represent PCR recombination events (hybrids) resulting from amplification of multiple but similar IGHV genes [42].

### In-depth IGHV analysis of BMPCs

**Figure 5:**
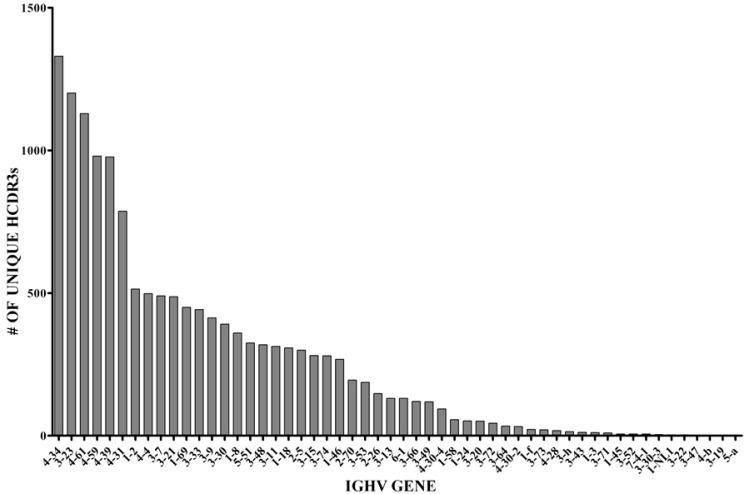
Number of unique HCDR3s and frequency of specific IGHV gene usage in normal BMPCs The sequences shown here represented 54 IGHV genes.

In another set of analyses, we studied in detail the IGHV usage of normal BMPCs using genomic DNA as the starting template. We also analyzed each IGHV gene for numbers of unique HCDR3 sequences (Figure 5).The IGHV repertoire pattern is more diverse than that of the presumably normal PCs present in the MM sample with 54 IGHV genes being represented as compared to 36. Of note, all 7 IGHV families are represented in both repertoires from the genomic DNA templates. Given that the IGHV6 and 7 families have few members, it is remarkable that we were able to detect these genes and is proof that our method of amplification captures all IGHV genes.

**Figure 6:**
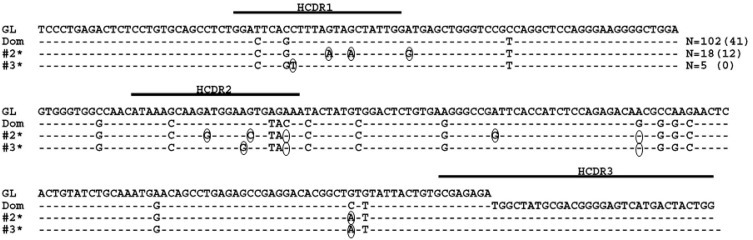
Normal BMPC clones with identical HCDR3s The largest clone, which used the IGHV3-7 gene (Dom), is shown here with putative subclones. Dashes indicate sequence homology with the IGHV3-7 GL sequence and nucleotide changes from the dominant clone are circled. Sequence numbers with an asterisk indicate that the nucleotide deviations from the dominant clone reflect reversions back to GL. Values in parentheses represent the number of identical sequences found in cDNA.

Using the same methodology described for the MM cohort we interrogated the normal BMPC IGHV sequence set for evidence of clonally related sequences and indeed, we found such sequences within the normal BMPCs. The largest clone utilized the IGHV3-7 gene with an HCDR3 amino acid sequence of ARDGYATGSHDY and included 102 identical sequences by both amino acid and nucleotide sequences with 5 N and P nucleotides in the HCDR3. We also observed two related sets of sequences, which consisted of 18 and 5 sequences that had the same mutation pattern and HCDR3 as the dominant sequence but differed in the IGHV region by 8 and 4 IGHV gene mutations, respectively (Figure 6). However, only set #2 met our criteria for verification (at least 10 sequences in both the DNA and cDNA cohort) and was therefore considered a subclone. Similar to what was observed in the MM patient, some of the nucleotide deviations between the subclones and the dominant clone were nucleotides that did not appear to mutate from the GL sequence.

### Characterization of somatic mutations in MM and BMPC clones and subclones

**Figure 7:**
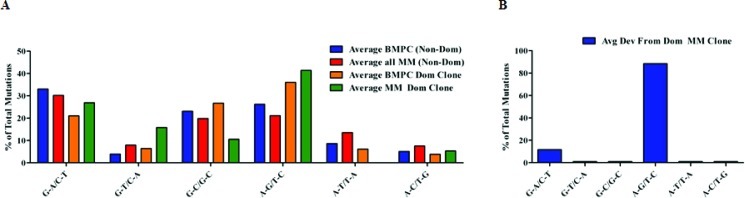
Characterization of mutations in BMPC and MM sequences For each sequence analyzed, nucleotide substitutions were counted, grouped into 6 complementary categories and represented as the percent of total mutations for that sequence. Groups of specific sequences were averaged and plotted. (A) The average expression of mutations in non-dominant (Non-Dom) BMPC sequences (n=10) and Non-Dom MM sequences (n=18; 8 from this pyrosequencing study and 10 from Sanger sequencing) were compared to the average expression of mutations in the dominant (Dom) BMPC and MM clones. (B) Characterization of the nucleotide substitutions exhibited by the putative MM subclones

We observed that each of the putative MM subclones displayed only single nucleotide substitutions from the dominant clone whereas clonally related BMPC sequences differed to an expected greater degree from each other. We therefore considered the possibility that some of the distinguishing nucleotide substitutions in the putative MM subclones instead resulted from *Taq* polymerase errors. To provide a baseline comparison for our analysis, we first determined the type of nucleotide substitutions exhibited by the dominant IGHV3-74 MM clone and the largest IGHV3-7 BMPC clone. We also carried out a similar analysis on at least 10 other (non-dominant) IGHV sequences from both the BMPC and MM sequence data sets as well as other MM patient samples previously subjected to Sanger sequencing from our tissue bank but not part of this detailed pyrosequencing study (Tschumper and Jelinek, unpublished data). Because a G to A change on one strand cannot be distinguished from a C to T occurring on the complementary DNA strand, we grouped the 12 possible nucleotide substitutions into 6 categories [43]. Thus, for each sequence analyzed, the 12 possible nucleotide substitutions were counted, grouped into 6 complementary categories and represented as the percent of total mutations for that sequence. When the average of percent mutations in the non-dominant BMPC and MM sequences are compared to the average percent mutations in the dominant BMPC and MM clonal sequences (and their respective subclones), there is evidence of bias towards transitions over transversions (Figure 7A). We next analyzed the type of nucleotide substitution displayed by each of the 44 putative MM subclones with nucleotide substitutions within the IGHV gene. As may be seen in Figure 7B, there was a notable overrepresentation of the transitions A-G/T-C in the putative MM subclones. Because it is known that *Taq* substitutions at A or T nucleotides are much more frequent than at G or C nucleotides [43] these results support a cautious interpretation of the significance of these MM subclones.

## DISCUSSION

In this study, we used massively parallel sequencing to investigate Ig repertoire profiles within BMPCs from a MM patient and from a control patient. Previous studies investigating MM IGHV sequences using labor intensive techniques concluded that MM cells do not display intraclonal IGHV sequence diversity. However, analysis was restricted to a small number of subclones (n=3-100, based on the specific study) [16, 17, 25-27] and these investigators did not definitively identify subclones of the dominant clone based on exact identity of the HCDR3. By contrast, our use of the Roche 454 GS-FLX Titanium chemistry allowed us to analyze approximately 30,000 Ig sequences increasing the likelihood of detecting rare subclones while also providing details regarding the Ig repertoire of BM resident non-malignant PCs in a MM patient. For comparison purposes, we also analyzed the Ig repertoire of normal BMPCs. Data sets of this magnitude pose analytic challenges, however, through use of previously published methodologies [31, 37] and development of our own algorithms, we were able to categorize sequences based on HCDR3 identity and IGHV gene usage allowing us to identify clonal sequences and putative subclones in both the MM and normal BMPC samples.

An initial global analysis verified that the genomic DNA samples yielded a more robust overall repertoire than did the cDNA samples on the basis of quantitating unique HCDR3s (Figure 1). Of the approximately 60 known IGHV genes in the IMGT/V-Quest reference sets (including functional genes and pseudogenes), 54 genes were identified in the BMPC DNA sample (Figure 5). Regarding similar analysis of non-tumor PCs in the MM sample, we did not identify a large number of non-IGHV3-74 non-productive clonal sequences. This observation suggests that the myeloma cells from this patient most likely express the second Ig heavy chain allele in a germline configuration. Despite the relatively low numbers of non-clonally related sequences available for analysis, it is striking that the few normal PCs coexisting with MM PCs remain quite diverse, i.e., 37 IGHV genes were represented in this data set. These results suggest that the MM clone is indiscriminate in suppressing the appearance of normal PCs. Although the IGHV gene usage appears to be less diverse than in a normal patient, our study design leaves open the possibility that with a larger sampling of normal BMPCs within a MM patient, we would have observed a higher level of diversity.

The primary advantage of our study design was our ability to rigorously assess the existence of PCs clonally related to the predominant MM clone. This is the largest reported dataset demonstrating that the vast majority of the malignant PCs in a MM patient exhibit an identical IGHV gene sequence and is consistent with the conclusions reached by other investigators following analysis of much smaller sequence cohorts [16, 17, 25-27]. This observation is also consistent with the idea that MM is a post-GC malignancy that can no longer undergo SHM. As a result of the depth of our sequence analysis, however, we also uncovered related sequences that were distinguished by a single nucleotide substitution whereas similar analysis of the clonally related normal BMPC sequences displayed multiple nucleotide substitutions. If these single nucleotide changes are genuine, it becomes unlikely that the MM-related subclones arose from the germinal center SHM process. Rather, the nucleotide deviations in MM may simply represent random somatic mutations occurring genome-wide in the tumor cell independent of the GC reaction, which deep sequencing allowed us to detect (Figure 3). Indeed, MM is believed to be a disease where considerable genetic evolution occurs. Whole genome sequencing of MM patients revealed unexpected somatic mutations across the MM genome as compared to normal DNA [44] and we have recently published evidence for ongoing double strand DNA breaks in primary MM cells [45].

However, it is also possible that some or all of the MM-related subclones instead reflect artifacts known to be problematic with this methodology. One type of artifact is insertions or deletions of the same nucleotide within homopolymer regions. By our algorithm, sequences with apparent insertions or deletions of the same nucleotide within homopolymers (defined as tracts of 4 or more of the same nucleotide) or substitutions of nucleotides within 2 base pairs of homopolymers were eliminated from consideration. These types of sequencing errors also may result in a low Phred score. Therefore by requiring a Phred score greater than 20, we eliminated errors due to homopolymers and other potential sequencing errors. We further required that a potential clonally-related sequence could only be designated as such if the precise sequence could be found a minimum of 10 times in the libraries generated from both cDNA and genomic DNA. Sequences meeting these criteria could be subdivided into 44 MM-related subclones, with each putative subclone exhibiting an identical HCDR3 region (VREDLSTSTWGFDY) and an IGHV3-74 gene with the same mutations as the dominant MM clone but with additional subclone-specific single point mutations in each sequence. An additional 4 putative subclones were discovered that expressed IGHV regions identical to the dominant clone but varied by a single nucleotide in the HDCR3 thus altering the HCDR3 amino acid sequence.

The second type of artifact associated with this methodology is *Taq* polymerase mediated nucleotide substitutions which involve errors at A or T nucleotides at a much greater rate than G or C nucleotides [43]. Because of this bias, we characterized the precise type of nucleotide substitution exhibited by each of the 44 putative MM subclones which expressed single nucleotide variances within the IGHV gene. Whereas normal BMPC sequences and MM clonal sequences obtained from a variety of patients contained nucleotide substitutions in all complementary categories, the single nucleotide deviations from the dominant clone in the putative subclones show a strong bias for G-A/C-T and A-G/T-C nucleotide substitutions (Figure 7B). Thus, almost 90% of the nucleotide changes were A-G or T-C substitutions, thereby calling into question whether all 44 subclones are *bona fide* subclones or whether some or all of them are artifacts resulting from *Taq* polymerase errors [31, 43, 46]. It should be noted that transition mutations are known to occur more frequently in general than transversion mutations due to nucleotide molecular structure [47] and we indeed observed this in our analysis of the somatic mutations displayed by normal BMPCs and MM samples (Figure 7A). Of interest, two previous studies of MM Ig clonality on a much smaller scale observed changes that were also mainly transitions of A-G and T-C and this was observed after multiple samplings and correcting for *Taq* errors [16, 27]. Thus, it is possible that some of the subclones described in our study are genuine. Support for this possibility is provided by considering the error rate of the *Taq* polymerase (2 x 10^−5^) used in our study in context with our sequencing depth of nearly 30,000 reads at an average read length of 350-bases per sample. Thus, we calculated that the first PCR error could happen between cycles 21 and 22, which would result in multiple false positive clones with the largest clone size of 128 reads. However, the largest observed clone size in our data was 56 indicating that the earliest possible PCR error cycle is 24. Based on this observation and that we required a minimal clone-size of 10 eliminating false positive clones from late-cycle PCR errors, we calculated that we would expect 7 false positive clones. With n=48 clones, the false positive rate in our data is estimated to be ~15%. Taken together with the observed bias for A-G or T-C substitutions in the subclones, we cautiously conclude that some of the clonally related sequences likely reflect genuine subclones. Further work using high fidelity *Taq* polymerase and serial samples obtained over time from MM patients is required to distinguish between these two possibilities. The latter approach would permit an assessment of whether the point mutations accumulated over time are a result of disease progression.

While numerous studies analyzing the repertoire and intraclonal heterogeneity in T and B cells using in-depth sequencing have been published, to our knowledge, massively parallel pyrosequencing has not yet been used to analyze IGHV sequences expressed by either normal or malignant BM resident PCs. Instead, the emphasis has been on high throughput screening of natural or synthetic antibody repertoires [34, 48, 49]. While our analysis was limited to one normal BMPC sample and one MM sample, it is apparent that only with massively parallel sequencing and meticulous analysis will evidence of MM intraclonal heterogeneity be discovered. Our study importantly describes in detail the utility and potential pitfalls of using this approach to study IGHV clonal diversity in MM patients and in normal BMPCs. Finally, we also demonstrate the importance of using genomic DNA as the starting material and we underscore the need for cautious interpretation of IGHV sequence data using this approach. Despite the noted caveats, this methodology holds great promise for estimating the level of B lineage clonal diversity in healthy adults during the aging process as well as in estimating the diversity of normal B lineage cells in patients with B lineage malignancies.

## METHODS AND MATERIALS

### Ethics Statement

Mayo Clinic Institutional Review Board (IRB) approval was obtained for collection of bone marrow from a MM patient and a normal donor. Patients provided written informed consent to collect tissue and investigation was conducted in accordance with the ethical standards of the Declaration of Helsinki.

A BM specimen was collected from the iliac crest of a 59 year old male with a confirmed diagnosis of untreated IgG lambda MM. At the time of collection, the patient had 40% PCs in the marrow with a plasma cell labeling index of 3.0% and an M-spike of 3.5 g/dl upon immunofixation. Fluorescence in situ hybridization revealed a variety of abnormalities, i.e., +3, +9, t(4;14), -17p, and -13.

Normal BM was collected from a 69 year old female undergoing orthopedic surgery for spinal stenosis. There was no history of hematologic malignancy or any other malignancy.

### Cell isolation

BM mononuclear cells obtained from both donors were isolated by Ficoll-Paque (GE Healthcare) density centrifugation. PCs were enriched by magnetic bead sorting using the Human CD138 Positive Selection kit (StemCell Technologies) with the Robosep instrument (StemCell Technologies) following the manufacturer’s protocol. The purity of both PC samples was >98% as assessed by morphology (Figure S2).

### RNA and DNA isolation, cDNA synthesis, IGHV PCR, and sequence analysis

RNA was isolated by the TRIzol method (Invitrogen) and 2 μg of RNA was converted to cDNA using the GE Healthcare First Strand Synthesis kit. Genomic DNA was also isolated from PCs using the 5’ Prime ArchivePure DNA Purification Kit (Fisher Scientific). Genomic DNA (200 ng) and cDNA (2 μl) were amplified using the Qiagen HotStarTaq MasterMix kit (Qiagen,) with Q solution (Qiagen) in a multiplex PCR reaction using 0.5 μM of each of 7 sense primers representing the 7 IGHV families [13, 50] in conjunction with 0.5 μM of antisense primer to the IGHJ region capable of amplifying all IGHJ genes. All primers had appropriate modifications for downstream emulsion PCR, amplicon capture and pyrosequencing (Table S4). Amplification was carried out in a Perkin Elmer 9600 thermocycler (Perkin Elmer) using the following conditions optimized for comprehensive amplification: 95°C for 15 min; 30 cycles of 95°C for 30 s, 60°C for 60 s, 72°C for 60 s; and a final cycle of 72°C for 10 min. Amplified products were visualized on a 1.5% agarose TAE gel, excised and purified with the Purelink Gel Extraction kit (Invitrogen). Purified MM BMPC products were directly sequenced on an ABI PRISM 3730xl DNA Analyzer (Applied Biosystems/Life Technologies). All MM and normal BMPC PCR products were subcloned (Topo-TA Cloning Kit, Invitrogen) with at least 48 clones from each sample analyzed to ensure that our PCR strategy could amplify IGHV genes from all 7 families. Resulting sequences were aligned to GL IGHV sequences using ImMunoGeneTics Information System reference sets and IMGT/V-Quest software (http://imgt.cines.fr) [51].

### Pyrosequencing and sequence analysis

Purified PCR products were processed for sequencing on a Roche 454 GS-FLX using the Titanium amplicon sequencing chemistry as described in the manufacturer’s protocol (Roche, 454 Life Sciences). The samples were run using 4 regions of an 8 region gasket and sequences were read from both the “A” and “B” primer ends.

Sequences that were shorter than the length distribution mode for each sample were filtered out and the remaining reads were aligned to GL IGHV sequences using IMGT/V-Quest software disallowing for insertions and deletions (indels) [51]. Non-Ig sequences or Ig sequences that were non-productive or did not have a complete HCDR3 were removed from the dataset.

After IGHV gene categorization and HCDR3 analysis, we used a customized in-house method similar to that used by Chen et al [37] to further interrogate the sequence data. Sequences were first grouped by IGHV gene categories and then by HCDR3 amino acid sequences. Those sequences with identical HCDR3s were further classified into subgroups with identical nucleotide sequence in the IGHV region.

To validate possible MM subclones resulting from intraclonal heterogeneity we followed an algorithm similar to that described by Campbell et al [31] to eliminate sequence artifacts caused by inherent errors in pyrosequencing. Clone size was defined as all sequences with an identical HCDR3 amino acid sequence and the same IGHV gene. Given the potential for extraordinary sequence diversity of IGHV genes and our analysis of only 24,000-36,000 IGHV sequences (see Table S1), it should be noted that ‘clone size’ in our studies is still an approximation. From this cohort, sequences with apparent insertions or deletions of the same nucleotide within homopolymers (defined as tracts of 4 or more of the same nucleotide) or substitutions of nucleotides within 2 base pairs of homopolymers were excluded from further analysis since these are more likely to be sequencing errors [52]. Of the remaining sequences, to be characterized as a MM-related subclone, we required the presence of 10 or more identical sequences in both the genomic DNA and cDNA-derived material, and both the forward and reverse orientations represented. Furthermore we required that all nucleotides within the sequence (including nucleotide changes in the subclones) had a Phred quality score greater than 20.

## Supplementary Tables and Figures










