# Supplementary material for: Comprehensive Assessment of Potential Multiple Myeloma Immunoglobulin Heavy Chain V-D-J Intraclonal Variation Using Massively Parallel Pyrosequencing
Source: Oncotarget. 2012 Apr 20;3(4):502–13. doi: 10.18632/oncotarget.469 (PMC3380583; doi:10.18632/oncotarget.469)
Supplement: Supplementary file 5 [file oncotarget-03-502-s005.pdf]

Comprehensive Assessment of Potential Multiple Myeloma Immunoglobulin Heavy Chain V-D-J Intraclonal Variation Using Massively Parallel Pyrosequencing-Tschumper et al

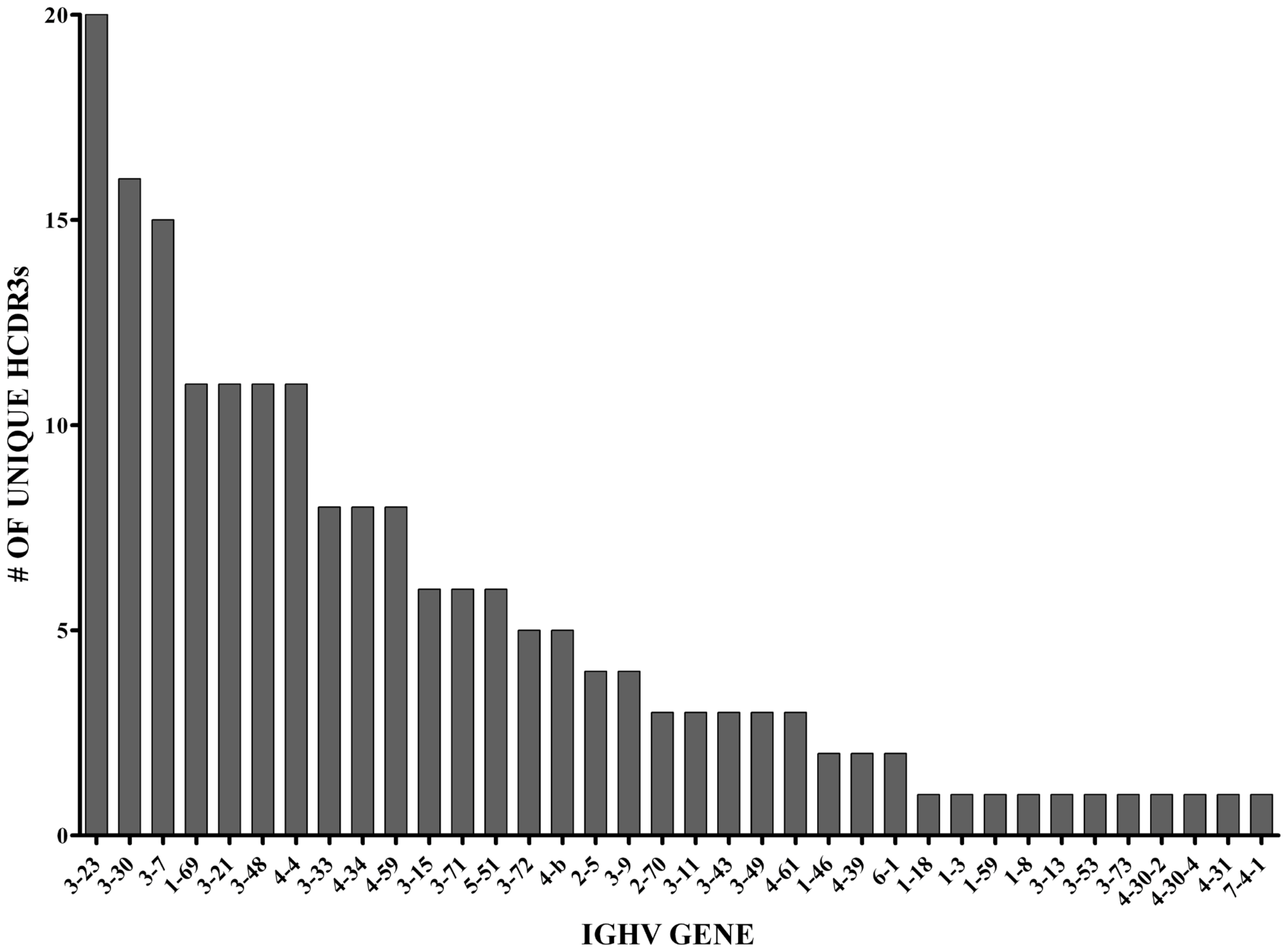

Figure S1: Distribution of IGHV genes other than the IGHV3-74 gene in MM DNA. Unique HCDR3s representing 36 other IGHV genes were found.

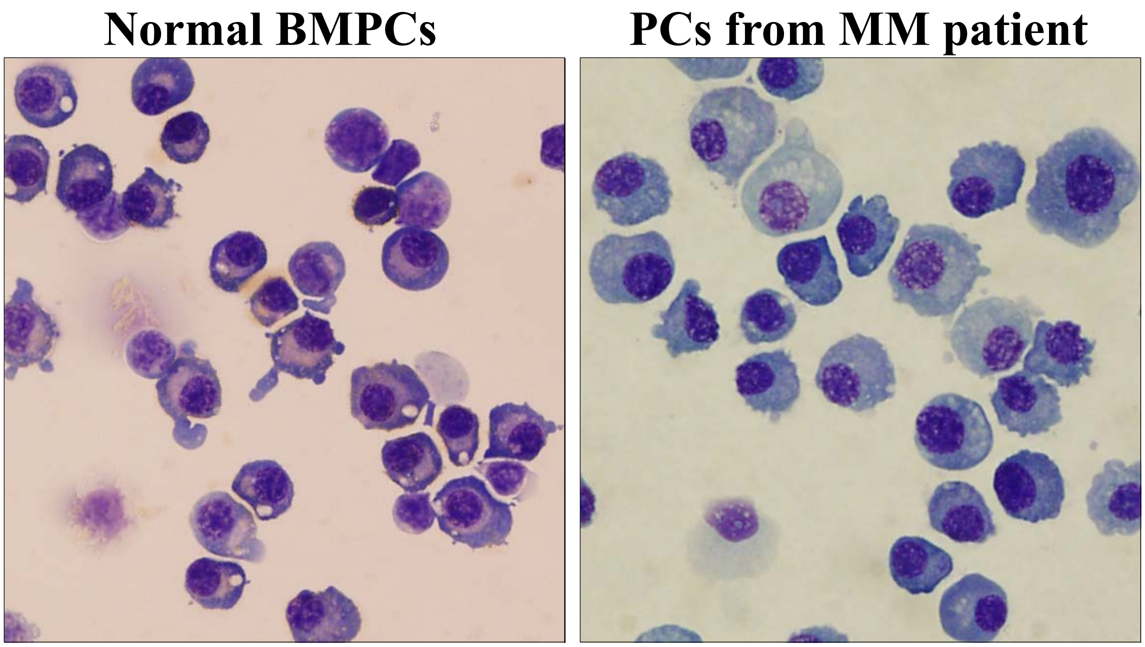

Figure S2: BMPCs from a normal surgical patient and a MM patient after CD138 bead purification. Post purification, cells were collected by cytospin centrifugation, stained with Wright's stain and photographed to show purity (60x magnification).
